# Levothyroxine Substitution in Patients with Subclinical Hypothyroidism and the Risk of Myocardial Infarction and Mortality

**DOI:** 10.1371/journal.pone.0129793

**Published:** 2015-06-12

**Authors:** Mette Nygaard Andersen, Anne-Marie Schjerning Olsen, Jesper Clausager Madsen, Jens Faber, Christian Torp-Pedersen, Gunnar Hilmar Gislason, Christian Selmer

**Affiliations:** 1 Department of Cardiology, Gentofte University Hospital, Hellerup, Denmark; 2 Elective Laboratory of the Capital Region, Copenhagen, Denmark; 3 Department of Endocrinology, Herlev University Hospital, Herlev, Denmark; 4 Faculty of Health Sciences, University of Copenhagen, Copenhagen, Denmark; 5 Department of Health Science and Technology, University of Aalborg, Aalborg, Denmark; 6 The Danish Heart Foundation, Copenhagen, Denmark; 7 The National Institute of Public Health, University of Southern Denmark, Copenhagen, Denmark; 8 Department of Internal Medicine, Amager University Hospital, Amager, Denmark; Rutgers University, UNITED STATES

## Abstract

**Background:**

Subclinical hypothyroidism is associated with a number of cardiovascular risk factors, yet only limited data exist on long-term outcome of levothyroxine treatment of this condition with respect to hard end-points. The aim of this retrospective cohort study was to determine effects of levothyroxine treatment on myocardial infarction (MI), cardiovascular death and all-cause mortality, in patients with subclinical hypothyroidism.

**Methods and Results:**

Primary care patients aged 18 years and older that underwent thyroid function tests between 2000 and 2009 were enrolled. Participants were identified by individual-level linkage of nationwide registers. Patients with subclinical hypothyroidism at baseline were included in the study. Exclusion criteria included a history of thyroid disease, related medication or medication affecting thyroid function. The total cohort comprised 628,953 patients of which 12,212 (1.9%) had subclinical hypothyroidism (mean age 55.2 [SD ± 18.8] years; 79.8% female). Within the first six months 2,483 (20.3%) patients claimed a prescription for levothyroxine. During a median follow-up of 5.0 (IQR: 5.2) years, 358 MI’s and 1,566 (12.8%) deaths were observed. Out of these, 766 of the deaths were cardiovascular related. No beneficial effects were found in levothyroxine treated patients on MI (IRR 1.08 [95% CI: 0.81 to 1.44]), cardiovascular death (IRR 1.02 [95% CI: 0.83 to 1.25]) or all-cause mortality (IRR 1.03 [95% CI: 0.90 to 1.19]), except in patients under the age of 65 years (IRR 0.63 [95% CI: 0.40 to 0.99]).

**Conclusion:**

Levothyroxine substitution in subclinical hypothyroid patients does not indicate an association with lower mortality or decreased risk of MI.

## Introduction

Subclinical hypothyroidism is biochemically defined as raised serum thyroid-stimulating hormone (TSH) concentrations with simultaneously occurring normal circulating thyroxine (FT4).[1] The condition is associated with a number of cardiovascular risk factors such as diastolic hypertension,[2, 3] weight gain,[4–6] insulin resistance,[7] hypercholesterolemia,[8] dyslipidaemia,[9] coronary heart disease[10, 11] and ischemic heart disease.[12–16] However, a number of questions still remain concerning the treatment of subclinical hypothyroidism with levothyroxine, and controversy exists regarding the management of the condition.[17–19] Current guidelines recommend that individuals under the age of 65 years with a serum TSH value above 10 mIU/L and symptoms of hypothyroidism should be treated with levothyroxine, but opinions regarding mild subclinical hypothyroidism (TSH ≤ 10 mIU/L) and patients over the age of 65 years vary.[20, 21] Some endocrinologists recommend that most patients with subclinical hypothyroidism, including those with a serum TSH value below 10 mIU/L, should be treated.[22]

The prevalence of subclinical hypothyroidism is approximately 4% in the general population, it is more common in females, and increases with age.[23] Roughly 3–5% of patients with subclinical hypothyroidism progress to overt hypothyroidism annually, with higher frequencies in persons with elevated thyroxine peroxidase autoantibody levels.[24] A clinical randomized trial is currently in the process of recruiting patients over the age of 65 years with subclinical hypothyroidism with the aim to investigate the effects of levothyroxine.[25]

Treatment of the condition remains controversial because it is still unknown whether intervention with substitution therapy is beneficial on hard endpoints such as cardiovascular disease and mortality.[26] This large register-based study was conducted to examine the hypothesis, that substitution treatment with levothyroxine in patients with subclinical hypothyroidism reduces the risk of myocardial infarction (MI), cardiovascular death and all-cause mortality.

## Methods

### Data sources

Each resident in Denmark is provided with a permanent and unique identification number which enables individual-level linkage between national administrative registers holding information on healthcare usage.[27] Five registers were used in this study: 1) The Danish National Patient Register, which has kept records of all hospital admissions since 1977. All admissions have been registered with one main discharge diagnosis, and, if applicable, one or more supplementary discharge diagnoses coded according to the International Classification of Diseases (ICD-8 until 1994 and from 1994 ICD-10).[28] Vital status was obtained from 2) The Civil Registration system, which records deaths for all Danish citizens.[27] Specific causes of death were obtained from 3) The Danish Register of Causes of Death.[29] Information on medication was obtained from 4) The Danish Register of Medicinal Product Statistics, which keeps records on all claimed prescriptions (coded according to the international Anatomical Therapeutic Chemical (ATC) Classification) from pharmacies in Denmark since 1994.[30] The register also holds information regarding quantity, strength, and date of dispensation as well as formulation and the affiliation of the physician issuing the prescription. Annual incomes were retrieved from 5) The Danish registers on personal income and transfer payments–The Danish Labour Market.[31] Socioeconomic status was defined by the average yearly gross household income in a 5-year period prior to inclusion in the study.

### The study population

The study cohort comprised citizens of Copenhagen over the age of 18, who at referral of their general practitioner underwent a thyroid function test at the Elective Laboratory of the Capital Region, Copenhagen, in the period between 1 January 2000 and 31 December 2009. TSH and FT4 were determined in serum by the commercially available ADVIA Centaur System (Bayer/Siemens, Tarrytown, NY). Patients with previous thyroid dysfunction i.e. previous prescriptions of thyroid hormones, anti-thyroid drugs or any thyroid-related hospital diagnoses were excluded from the study. Patients were categorized according to their thyroid status at the time of their first thyroid function test by traditional definitions of thyroid dysfunction (Table 1).

**Table 1:** Definitions of thyroid disease and thyroid dysfunction levels.

Thyroid dysfunction definitions	TSH	FT4
Euthyroidism	0.2–5.0 mlU/L	9–22 pmol/L
Subclinical Hypothyroidism	> 5.0 mlU/L	9–22 pmol/L
Grade I	5.0–10 mIU/L	9–22 pmol/L
Grade II	> 10 mIU/L	9–22 pmol/L

Subclinical hypothyroidism was categorized in two subdivisions: Grade I with mildly increased TSH levels (5.0–10.0 mlU/l) and Grade II with severely increased TSH levels (> 10.0 mIU/l).[32, 33] Furthermore, patients were divided into above and below the age of 65 years.

### Study design

The study was a register-based retrospective cohort study of patients with subclinical hypothyroidism in the period 2000–2009. Patients were divided into groups of untreated and treated defined as those who initiated levothyroxine treatment within six months from the date of their first thyroid function test. This definition was chosen to ensure that the thyroid function test and subsequent prescription of levothyroxine had a causal relationship. Patients entered the study at the time of their thyroid function test. The treated patients contributed with risk time in the untreated group until they initiated levothyroxine treatment, at which they were moved from the untreated group to the treated group. Patients left the study at death, emigration, or end-of-study.

### Comorbidity and concomitant medical therapy

Comorbidities such as ischemic heart disease, heart failure, MI and stroke were identified from The Danish National Patient Register (Table 2).

**Table 2:** Diagnoses (ICD-8 and ICD-10) and medication (ATC) codes used in the study.

Diseases & medication	ICD & ATC codes
Thyroid diseases and medication	
Hypothyroidism	ICD-10: E02-03, E063
Hyperthyroidism	ICD-10: E05, E062
Any thyroid related disease	ICD-8: 240–246, ICD-10: E00-E06, O905
Levothyroxine	ATC: H03AA01
Methimazole	ATC: H03BB01, H03BB02
Propylthiouracil (PTU)	ATC: H03BA02
Cardiovascular disease and medication	
Ischemic heart disease	ICD-8: 411–414, ICD-10: I20, I23-I2
Myocardial Infarction (MI)	ICD-8: 410, ICD-10: I21, I22
Any cardiovascular related diagnoses	ICD-10: I00-I99
Amiodarone	ATC: C01BD01
Other diseases and medication	
Lithium	ATC: N05AN01
Corticosteroids	ATC: H02AB

Charlson Comorbidity Index was calculated on basis of pre-specified diagnoses up to five years prior to cohort entry.[34, 35] Medication known to affect thyroid function, such as thyroid hormones, anti-thyroid medications, lithium, amiodarone, and glucocorticoids,[36, 37] were identified from The Danish Register of Medicinal Product Statistics.

### Dose and duration of treatment

The Danish Register of Medical Product Statistics does not hold information regarding prescribed daily dosage of the medication. Therefore, we estimated the daily dosage when each new prescription was dispensed, by calculating the average dosages from up to seven consecutive prescriptions. This algorithm allowed the dosage to change when a new prescription was dispensed. This method, used to determine the average treatment time and dose has previously been described.[38, 39]

### Outcomes

The primary outcome of interest was all-cause mortality, with the secondary outcomes being MI, including fatal and non-fatal MI, and cardiovascular death, determined as death caused by any diseases or conditions related to the cardiovascular system (Table 2). The MI diagnoses reported to the registers have been validated and found reliable with a sensitivity of 91% and a positive predictive value of 93%.[40]

### Statistical analysis

Baseline characteristics are presented as numbers with percentages for categorical variables and as means ± standard deviations (SD) for continuous variables. Median follow-up time and average treatment time is reported with interquartile range (IQR). The incidence rates (IRs) were calculated as number of events per 1000 person-years (py) stratified by levothyroxine substitution, age as a continuous variable and two grades of subclinical hypothyroidism. Poisson regression models were constructed to estimate incidence rate ratios (IRRs, with 95% confidence intervals [CIs]) for the outcomes. Poisson regression models were adjusted for age, gender and Charlson Comorbidity Index and therefore included two time scales: calendar time with bands split in 1-year periods after 1 January 2000 and duration since the first thyroid function testing. Age was calculated at the beginning of each interval. Individuals were censored at the time of fatal or non-fatal event, at emigration or end of study (31 December 2009). A 95% significance level was used in all analyses including the interactions testing.

A number of sensitivity analyses were performed to validate the primary findings. First, we adjusted the main model for ischemic heart disease and socioeconomic status at baseline. Second, we did an identical sensitivity analysis using only patients with a Charlson Comorbidity Index equal zero, corresponding to no known comorbidities. Third, run-in periods of three, nine and twelve months from the time of the first thyroid function test until the time of the first prescription of levothyroxine were used. Fourth, time was divided into smaller intervals in order to ensure a constant rate in time division (bands were split every three- and six months from 1 January 2000). Finally, we obtained test results for a second thyroid function test, within 3 months from the first, in order to verify the thyroid function status of the patient group initially classified with subclinical hypothyroidism.

All statistical analyses were performed with the SAS Statistical Software package version 9.2 (SAS Institute Inc., Gary, NC, USA) and Stata Software version 11 (StataCorp, College Station, TX, USA).

### Ethics

The Danish Data protection Agency approved this study (Ref. No. 2007-58-0015/GEH-2014-018; I-Suite No. 02736) and data were made available for this study in an anonymised format preventing identification of individuals. Retrospective studies do not require ethical approval in Denmark.

## Results

**Fig 1:**
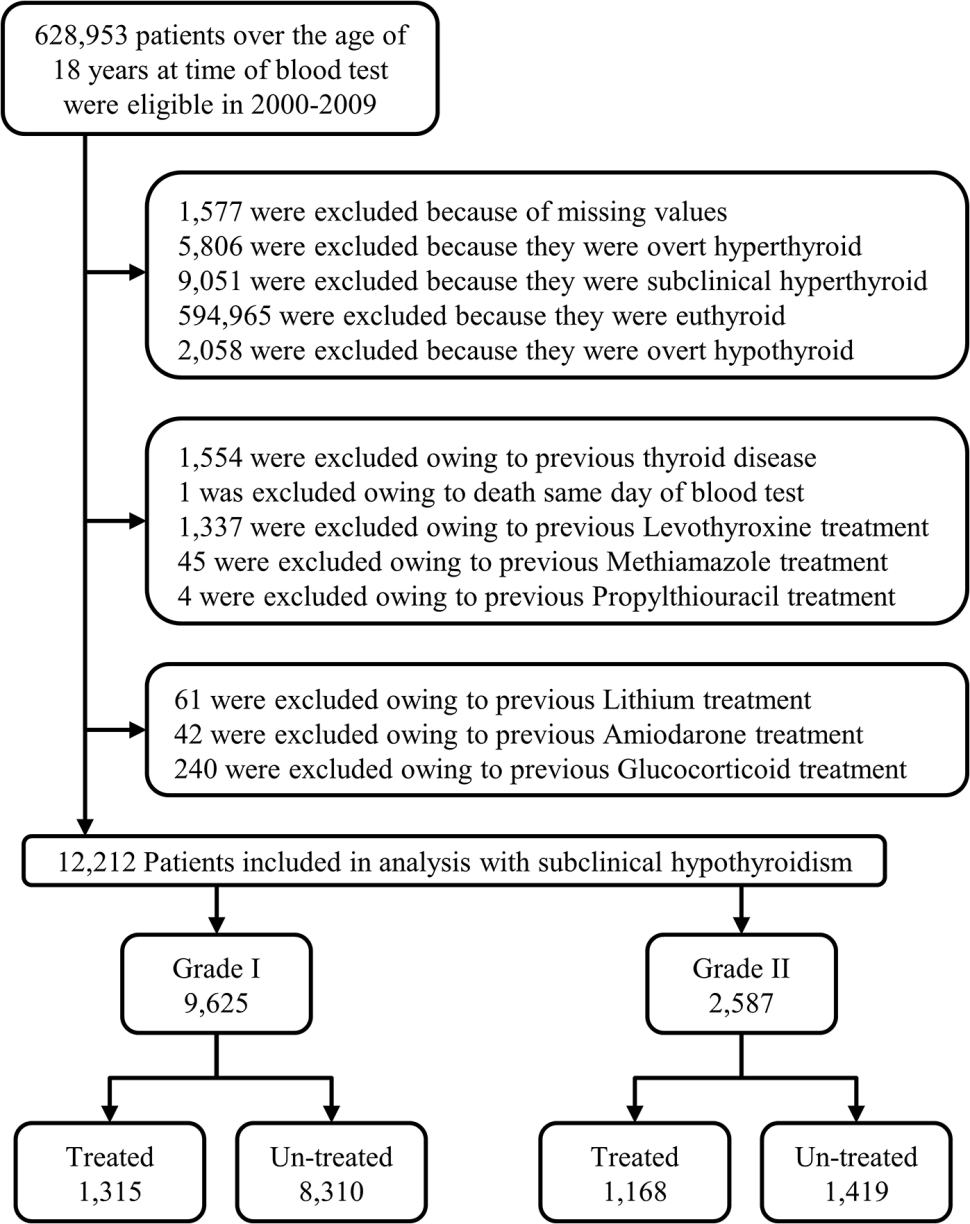
Flowchart. Selection of the study population.

A total of 628,935 individuals were included at first-time thyroid function test out of which 12,212 (1.9%) were classified with subclinical hypothyroidism. Selection of the study cohort is illustrated in Fig 1 and the baseline characteristics of the cohort are presented in Table 3.

**Table 3:** Baseline characteristics.

	Subclinical Hypothyroidism		Subclinical Hypothyroidism		Total Population
	Grade I		Grade II		
	Treated	Un-treated	Treated	Un-treated	
	*(n = 1*,*315)*	*(n = 8*,*310)*	*(n = 1*,*168)*	*(n = 1*,*419)*	*(n = 12*,*212)*
Age (±SD), years					
Mean age	53.0 (17.3)	55.3 (19.2)	54.4 (17.5)	57.0 (18.2)	55.2 (18.8)
Min.-max. age	18–96	18–102	20–99	18–100	18–102
Mean age, Women	52.4 (17.8)	55.7 (19.3)	54.5 (17.6)	57.1 (18.4)	55.4 (18.9)
Mean age, Men	57.0 (17.4)	54.1 (18.7)	53.6 (17.0)	56.6 (17.8)	54.5 (18.4)
Sex, No. (%)					
Women	1141 (86.8)	6457 (77.7)	1004 (86.0)	1141 (80.4)	9,743 (79.8)
Men	174 (13.2)	1853 (22.3)	164 (14.0)	278 (19.6)	2,469 (20.2)
Thyroid Function, median (IQR)					
TSH, mIU/L	7.3	6.2	17.0	14.0	6.9
	(6.2–8.7)	(5.5–7.4)	(13.0–25.0)	(12.0–18.0)	(5.7–9.7)
FT4, pmol/L	12.3	13.5	11.0	11.9	13.0
	(11.1–13.6)	(12.2–15.0)	(10.0–12.2)	(10.8–13.2)	(11.5–14.5)
Comorbidity, No (%)					
Ischemic heart disease	56 (4.3)	551 (6.6)	57 (4.9)	90 (6.3)	750 (6.1)
Heart Failure	25 (1.9)	263 (3.2)	17 (1.5)	39 (2.7)	344 (2.8)
Stroke	23 (1.7)	250 (3.0)	32 (2.7)	45 (3.2)	350 (2.9)
Diabetes	49 (3.7)	324 (3.9)	31 (2.7)	55 (3.9)	459 (3.8)
Myocardial Infarction	24 (1.8)	248 (3.0)	30 (2.1)	42 (3.0)	344 (2.8)
Charlson Comorbidity index No. (%)					
0	1,228 (93.4)	7,705 (92.7)	1,093 (93.6)	1,323 (93.2)	11,349 (93)
1	44 (3.4)	295 (3.5)	36 (3.1)	52 (3.7)	427 (3.5)
2	28 (2.1)	212 (2.6)	34 (2.9)	34 (2.4)	308 (2.6)
3+	15 (1.1)	98 (1.2)	5 (0.4)	10 (0.7)	128 (1.0)
Yearly income on quintiles, No. (%)					
0 (lowest)	150 (11.4)	1,145 (13.8)	109 (9.3)	151 (10.6)	1,555 (12.7)
1	263 (20.0)	2,168 (26.1)	259 (22.2)	408 (28.7)	3,098 (25.6)
2	282 (21.5)	1,841 (22.1)	257 (22.0)	332 (23.4)	2,712 (22.1)
3	333 (25.3)	1,602 (19.3)	265 (22.7)	276 (19.5)	2,476 (20.2)
4 (highest)	287 (21.8)	1,554 (18.7)	278 (23.8)	252 (17.8)	2,371 (19.4)

The study population comprised mainly women (79.8%) and the mean age was 55.2 years (SD ± 18.8). The treated patient group had slightly less comorbidity and was a few years younger with the exception of men with Grade I. Otherwise, the two groups had no major distinctions in health.

Within the first six months from the date of thyroid function test, 2,483 (20.3%) patients claimed prescriptions for levothyroxine. Out of these, 8% were treated for a maximum of 30 days, 6% were treated between 31 and 180 days, 7% were treated between 181 and 365 days, 13% were treated for 1–2 years, 13% were treated for 2–3 years and 54% were treated for more than 3 years. The average treatment time was 1,355 (IQR: 1,636) days and the average daily dosage prescribed was 79.7 (SD ± 30.8) micrograms.

The 9,729 patients defined as untreated either initiated levothyroxine treatment later than six months after their initial thyroid function test (3,799, corresponding to 39.0% of the original 9,729), or did not initiate any substitution treatment (5,930 corresponding to 61% of the original 9,729).

During a median follow-up of 5.0 (IQR: 5.2) years, 358 myocardial infarctions were registered, and a total of 1.566 (12.8%) patients died. Out of these deaths 766 were cardiovascular related.

Some 205 patients were lost to follow-up due to emigration and were censored.

### All-cause mortality

The unadjusted incidence rates for all-cause mortality in subclinical hypothyroidism were 26.4/1000 py for the untreated group and 22.3/1000 py for the treated group. Considering patients with Grade I the unadjusted incidence rates were 26.7/1000 py and 21.8/1000 py for the untreated and treated groups respectively while the unadjusted incidence rates for Grade II were 24.9/1000 py and 22.9/1000 py for the untreated and treated groups respectively.

**Fig 2:**
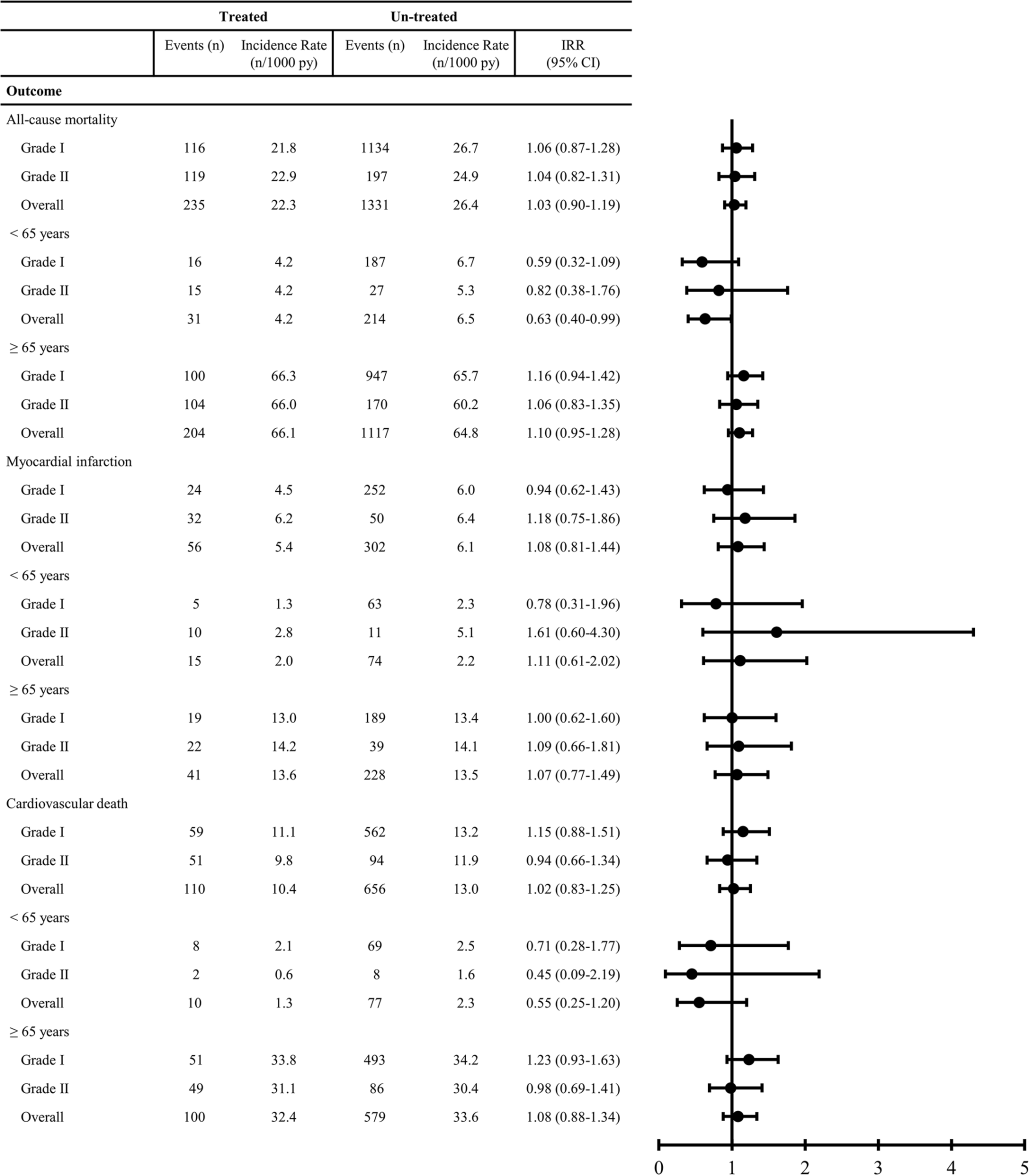
Incidence Rates (IRs) and Incidence Rate Ratios (IRR). Stratified on treatment, two grades of subclinical hypothyroidism, age and adjusted for gender, age, Charlson Comorbidity Index and calendar year. CI = Confidence Interval, py = person-years.

The adjusted Incidence Rate Ratios did not show any influence of treatment for neither the overall group of patients with subclinical hypothyroidism (IRR 1.03 [95% CI: 0.90 to 1.19]), nor the patients with Grade I (IRR 1.06 [95% CI: 0.87 to 1.28]), nor Grade II (IRR 1.04 [95% CI: 0.82 to 1.31]), see Fig 2.

When patients were divided into age groups, the results for overall subclinical hypothyroidism showed that treated patients under the age of 65 years had a significant lower mortality when compared to untreated patients under the age of 65 years (IRR 0.63 [95% CI: 0.40 to 0.99]). When considering patients under the age of 65 years with Grade I (IRR 0.59 [95% CI: 0.32 to 1.09]) and Grade II (IRR 0.82 [95% CI: 0.38 to 1.76]) isolated, this finding was no longer significant. The results for patients over the age of 65 years were similar to the main results, see Fig 2.

### Myocardial infarction

The unadjusted incidence rates for myocardial infarction in subclinical hypothyroidism were 6.1/1000 py for the untreated group and 5.4/1000 py for the treated group. Considering patients with Grade I the unadjusted incidence rates were 6.0/1000 py and 4.5/1000 py for the untreated and treated groups respectively while the unadjusted incidence rates for Grade II were 6.4/1000 py and 6.2/1000 py for the untreated and treated groups respectively.

The adjusted Incidence Rate Ratios did not show any influence of treatment for neither the overall group of patients with subclinical hypothyroidism (IRR 1.08 [95% CI: 0.81 to 1.44]), nor the patients with Grade I (IRR 0.94 [95% CI: 0.62 to 1.43]), nor Grade II (IRR 1.18 [95% CI: 0.75 to 1.86]). Grouping by age yielded no significant differences in the main results, see Fig 2.

### Cardiovascular death

The unadjusted incidence rates for cardiovascular death in subclinical hypothyroidism were 13.0/1000 py for the untreated group and 10.4/1000 py for the treated group. Considering patients with Grade I the unadjusted incidence rates were 13.2/1000 py and 11.1/1000 py for the untreated and treated groups respectively while the unadjusted incidence rates for Grade II were 11.9/1000 py and 9.8/1000 py for the untreated and treated groups respectively.

The adjusted Incidence Rate Ratios did not show any influence of treatment for neither the overall group of patients with subclinical hypothyroidism (IRR 1.02 [95% CI: 0.83 to 1.25]), nor the patients with Grade I (IRR 1.15 [95% CI: 0.88 to 1.51]), nor Grade II (IRR 0.94 [95% CI: 0.66 to 1.34]). Grouping by age yielded no significant differences in the main results, see Fig 2.

### Sensitivity analyses

Adjusting the main model for ischemic heart disease and socioeconomic status at baseline did not produce any significant impact on the results obtained with the main model nor did including only patients with Charlson Comorbidity Index equal zero. The gender-stratified analysis showed no difference from our main results, with one exception where it appeared that levothyroxine treated men classified with Grade I had a higher risk of cardiovascular death (17 events among treated and 137 events among untreated) when compared to untreated men in the same classification (IRR 1.69 [95% CI: 1.02 to 2.80]).

Using different run-in periods between thyroid function test and initiation of levothyroxine (three, nine and twelve months) did not affect the main results. Also, the main results were not altered when time was divided into smaller intervals.

Out of 12,212 patients initially classified with subclinical hypothyroidism, 3,443 (28.2%) had a second thyroid function test within three months from the first test. Out of these, 1.675 (48.6%) patients were confirmed subclinical hypothyroid. Conducting the same analysis for this group yielded no differences from the main results, see Table 4. Interactions of levothyroxine treatment with age and gender were examined, and no clinical important interactions were found.

**Table 4:** Sensitivity analyses.

	Subclinical hypothyroidism		
**All-cause mortality**	**Grade I**	**Grade II**	**Overall**
Main results	1.06 (0.87–1.28)	1.04 (0.82–1.31)	1.03 (0.90–1.19)
Adjusted for ischemic heart disease at baseline	1.05 (0.64–1.72)	0.79 (0.42–1.47)	0.96 (0.66–1.39)
Adjusted for socioeconomic status at baseline	1.05 (0.87–1.27)	1.05 (0.83–1.33)	1.03 (0.90–1.19)
Individuals with no known comorbidities	1.08 (0.87–1.34)	1.19 (0.92–1.54)	1.13 (0.96–1.32)
Stratified by gender			
Women	0.97 (0.78–1.21)	1.02 (0.79–1.31)	0.99 (0.85–1.16)
Men	1.42 (0.97–2.07)	1.17 (0.61–2.24)	1.24 (0.89–1.71)
Time to first prescription			
3 months	0.96 (0.76–1.20)	0.99 (0.78–1.26)	0.96 (0.82–1.13)
9 months	1.01 (0.84–1.22)	1.08 (0.86–1.36)	1.02 (0.90-1-17)
12 months	1.03 (0.87–1.23)	1.10 (0.88–1.38)	1.04 (0.91–1.18)
Stratified by follow-up time			
3 months	1.06 (0.87–1.28)	1.03 (0.82–1.31)	1.03 (0.90–1.19)
6 months	1.06 (0.87–1.28)	1.03 (0.82–1.31)	1.03 (0.90-1-19)
Using only patients with diagnoses confirmed in a second thyroid function test	-	-	0.90 (0.66–1.22)
**MI**	**Grade I**	**Grade II**	**Overall**
Main results	1.66 (0.70–3.95)	1.45 (0.49–4.31)	1.42 (0.76–2.68)
Adjusted for ischemic heart disease at baseline	0.86 (0.40–1.87)	0.81 (0.36–1.84)	0.89 (0.53–1.51)
Adjusted for socioeconomic status at baseline	0.94 (0.62–1.43)	1.21 (0.77–1.90)	1.08 (0.81–1.44)
Individuals with no known comorbidities	0.93 (0.57–1.51)	1.07 (0.66–1.73)	1.09 (0.79–1.51)
Stratified by gender			
Women	0.84 (0.50–1.41)	1.05 (0.63–1.78)	0.98 (0.70–1.38)
Men	1.21 (0.58–2.51)	1.66 (0.70–3.92)	1.41 (0.83–2.40)
Time to first prescription			
3 months	1.70 (0.71–4.05)	2.40 (0.78–7.40)	1.68 (0.89–3.16)
9 months	1.55 (0.69–3.46)	1.44 (0.49–4.29)	1.36 (0.74–2.51)
12 months	1.34 (0.60–2.99)	1.44 (0.49–4.29)	1.27 (0.69–2.34)
Stratified by follow-up time			
3 months	1.65 (0.69–3.95)	1.45 (0.49–4.32)	1.42 (0.76–2.68)
6 months	1.66 (0.69–3.95)	1.45 (0.49–4.31)	1.42 (0.76–2.68)
Using only patients with diagnoses confirmed in a second thyroid function test	-	-	0.72 (0.39–1.34)
**CV Death**	**Grade I**	**Grade II**	**Overall**
Main results	1.15 (0.88–1.51)	0.94 (0.66–1.34)	1.02 (0.83–1.25)
Adjusted for ischemic heart disease at baseline	1.09 (0.59–2.03)	1.05 (0.51–2.14)	1.15 (0.74–1.78)
Adjusted for socioeconomic status at baseline	1.14 (0.87–1.49)	0.93 (0.65–1.33)	1.01 (0.83–1.24)
Individuals with no known comorbidities	1.20 (0.88–1.62)	1.03 (0.70–1.51)	1.11 (0.88–1.39)
Stratified by gender			
Women	1.02 (0.74–1.40)	0.97 (0.67–1.41)	0.96 (0.77–1.21)
Men	1.69 (1.02–2.80)*	0.78 (0.26–2.27)	1.32 (0.83–2.08)
Time to first prescription			
3 months	1.10 (0.80–1.49)	0.82 (0.56–1.19)	0.93 (0.74–1.17)
9 months	1.06 (0.82–1.38)	1.00 (0.71–1.40)	0.99 (0.82–1.21)
12 months	1.08 (0.84–1.38)	1.08 (0.77–1.51)	1.03 (0.85–1.24)
Stratified by follow-up time			
3 months	1.15 (0.88–1.51)	0.94 (0.66–1.34)	1.02 (0.83–1.25)
6 months	1.15 (0.88–1.51)	0.94 (0.66–1.34)	1.02 (0.83–1.25)
Using only patients with diagnoses confirmed in a second thyroid function test	-	-	0.89 (0.58–1.38)

## Discussion

In this large cohort study no association between levothyroxine treatment and risk of myocardial infarction (MI) and mortality was found, except in younger patients where it may seem that levothyroxine has a marginal protective effect on all-cause mortality.

Previous cohort studies on subclinical hypothyroidism and hard endpoints have been conflicting with some reporting increased longevity[16, 41] and some finding no association.[42, 43] Interestingly, a recent cohort study by Razvi et al. on 4,735 primary care patients in the UK found fewer ischemic heart disease events and reduced all-cause mortality in younger (40–70 years) subclinical hypothyroid patients substituted with levothyroxine.[44] The findings in our study, which show that levothyroxine has a marginal protective effect on all-cause mortality on patients under the age of 65 years, are partly in keeping with those of Razvi et al.. A similar protective effect could not be demonstrated when considering MI and cardiovascular death. Neither was the protective effect on all-cause mortality in younger patients significant when stratifying on Grade I and Grade II subclinical hypothyroidism. On the other hand, one sensitivity analysis showed that levothyroxine treated men classified with Grade I subclinical hypothyroidism had a higher risk of cardiovascular death. One explanation for this finding may be that we studied a small group (n = 174) with slightly more cardiovascular comorbidities and therefore might observe negative side effects of levothyroxine on the cardiovascular system. Another possibility is that this finding might be a chance finding, since it was not possible to recreate the same association considering MI and all-cause mortality. The above mentioned results from the study by Razvi et al. is also in keeping with a large meta-analysis of twelve randomized controlled trials, comparing thyroid hormone treatment with placebo in 350 participants with subclinical hypothyroidism made by Villar et al.[45].

Although subclinical hypothyroidism is associated with a number of well-known cardiovascular risk factors, we could not demonstrate an effect on the risk of MI and cardiovascular death in patients treated with levothyroxine. This was unexpected, as several studies have shown that levothyroxine decreases total plasma cholesterol in patients with hypercholesterolemia and subclinical hypothyroidism,[8, 46] Although we were not able to demonstrate a beneficial effect on all-cause mortality, MI and cardiovascular death in these patients, it is important to remember that there may be other benefits of treating subclinical hypothyroidism such as e.g. physical wellbeing and mitigation of general fatigue.[47, 48]

The potential harmful effects of treatment (and overtreatment) with levothyroxine are well-known and include cardiovascular and skeletal side effects.[49, 50] A clinical case-control study by Biondi et al. involving twenty patients substituted with levothyroxine and twenty normal controls, demonstrated an increased risk of cardiac side-effects, such as tachycardia and atrial arrhythmias, in the patients substituted with suppressive doses of levothyroxine.[49] A Danish meta-analysis of thirteen cross-sectional studies by Faber and Galløe[51] showed reduction in bone mass due to reduced serum TSH owing to levothyroxine treatment in postmenopausal women. Another study by Ross et al. involving 28 premenopausal women prescribed with levothyroxine found a 9% reduction in bone density compared with normal premenopausal age-matched controls.[50] These findings might bring concern that levothyroxine treatment, suppressing serum TSH, might promote osteoporosis. Studies have shown that around 40–48% of substituted hypothyroid patients have abnormal TSH levels. [52, 53] The Colorado Survey showed that out of 1,525 patients substituted with levothyroxine, 17.6% had subclinical hypothyroidism and 20.7% had subclinical hyperthyroidism.[52] This finding is in keeping with an observational study by Parle et al., which found that in 146 patients treated with levothyroxine, 21% were over-treated.[53] These data suggest that there exists a tendency towards overtreatment with levothyroxine, which may lead to both cardiac- and skeletal side effects, but also weight loss, which patients may welcome.[54]

International guidelines recommend considering treatment of subclinical hypothyroidism when TSH > 10 mIU/L (except in elderly patients). For patients with TSH ≤ 10 mIU/L the treatment should be planned on an individual level, mainly based on symptomatology.[20, 21, 55] Looking at the patients with Grade II subclinical hypothyroidism (TSH > 10 mIU/L) in this present study, less than half of these patients are treated, even though the current guidelines recommend treatment. This may reflect the uncertainty and disagreement among doctors behind the decision to initiation of treatment.

The overall main results in our study partly support the current guidelines, which state that treatment decisions should be kept on an individual level and always consider the symptomatology and comorbidities of the individual patient. Given the fact that this study shows no convincing overall influence of levothyroxine on the risk of MI, cardiovascular death and all-cause mortality, the decision to treat a patient should primarily be based on a desire to relieve symptoms.

### Strengths and limitations of the study

The large cohort and the availability of complete follow-up data are the main strengths of this study. Specifically, we had access to thyroid function tests from approximately half of the individuals in the capital region of Denmark and the use of levothyroxine and outcomes represent real-world data from primary care.

It is essential to notice that we did not have any knowledge of the reasons for thyroid function testing, how the patients were monitored while undergoing treatment or how the patients’ hormonal levels responded to treatment i.e. whether treated patients became euthyroid or remained subclinical hypothyroid. Therefore, the possibility that the results were influenced by selection bias cannot be excluded. Furthermore, the selection of subjects for thyroid function testing by physicians could in itself be associated with the studied outcomes of interest. Additionally, we did not have access to a number of relevant clinical information such as blood pressure, BMI, serum lipid, smoking status, electrocardiography, or the specific cause of thyroid dysfunction, as we do not hold information regarding radioiodine treatment or thyroid autoantibody levels. However, a previous similar study did not found that BMI, smoking status, total cholesterol or blood pressure, were important confounders.[44]

This study has a relatively short mean follow-up period of 5.0 years and it should be acknowledged that the outcomes studied might occur after longer periods of thyroid disease and levothyroxine treatment.

The results of this study could be explained by the fact that patients with subclinical hypothyroidism may experience spontaneous normalization of TSH values and regress to euthyroidism without treatment, which could underestimate the examined endpoints. On the other hand, some patients with subclinical hypothyroidism might progress to overt hypothyroidism, which could overestimate the risk in this study.[56] Another limitation is the definition of the treated patient group. The six-month run-in period from the first thyroid function test in which the patients could initiate treatment with levothyroxine was chosen to make certain that the subsequent treatment was associated with the specific thyroid function test and not initiated due to e.g. overt hypothyroidism developed during the follow-up period. This means that patients who initiated treatment after six months were not considered in the treated group. However, sensitivity analyses using three, nine and twelve month run-in periods did not change the main results. In individuals with subclinical hypothyroidism who initiated substitution treatment within the first 6 months the average dose of levothyroxine was estimated to 80 micrograms/day, which seems reasonable, considering that 100–150 micrograms pr. day are often needed in overt hypothyroidism.[57]

It was not possible to verify the thyroid function status of all patients due to incomplete data, but an analysis using only patients with confirmed subclinical hypothyroidism by control blood tests showed no significantly changes in the main results.

A confounder which could affect the results might be the fact that doctors could be more likely to prescribe treatment to patients with inability to follow a healthy lifestyle such as those with overweight, smoking and physical inactivity. If that is indeed the case, the risks associated with the patients’ basic lifestyle might suppress the hypothesized beneficial effect of treatment.

The study included only individuals in primary care setting, and therefore extrapolation of these results to patients from in- or outpatient clinics should be performed with caution. Likewise, extrapolation of these results to other ethnic groups than Caucasians should be done with care since the Danish population comprises mainly this group.

There may be other health benefits of treating subclinical hypothyroidism, such as changes in cholesterol levels, symptom relief, weight loss, or quality of life in general, which this study does not consider. Treatment may well have a positive effect on the above parameters, which in itself can have a significant impact on the patient. Patient-based decisions with consideration of the individual history should be made when starting treatment with levothyroxine, as the goals of treatment are not necessarily the outcomes examined in this study.

### Conclusion

In conclusion, substitution treatment with levothyroxine in patients with subclinical hypothyroidism, is not associated with lower mortality or decreased risk of myocardial infarction. Clinical randomized controlled trials are needed to confirm the findings of this study.
